# Glycine Transporter 2: Mechanism and Allosteric Modulation

**DOI:** 10.3389/fmolb.2021.734427

**Published:** 2021-11-05

**Authors:** Zachary J. Frangos, Ryan P. Cantwell Chater, Robert J. Vandenberg

**Affiliations:** Transporter Biology Group, School of Medical Sciences, Faculty of Medicine and Health, University of Sydney, Sydney, NSW, Australia

**Keywords:** glycine transporter, allosteric modulation, solute carrier 6 (SLC6), neurotransmitter sodium symporters (NSS), lipid modulation

## Abstract

Neurotransmitter sodium symporters (NSS) are a subfamily of SLC6 transporters responsible for regulating neurotransmitter signalling. They are a major target for psychoactive substances including antidepressants and drugs of abuse, prompting substantial research into their modulation and structure-function dynamics. Recently, a series of allosteric transport inhibitors have been identified, which may reduce side effect profiles, compared to orthosteric inhibitors. Allosteric inhibitors are also likely to provide different clearance kinetics compared to competitive inhibitors and potentially better clinical outcomes. Crystal structures and homology models have identified several allosteric modulatory sites on NSS including the vestibule allosteric site (VAS), lipid allosteric site (LAS) and cholesterol binding site (CHOL1). Whilst the architecture of eukaryotic NSS is generally well conserved there are differences in regions that form the VAS, LAS, and CHOL1. Here, we describe ligand-protein interactions that stabilize binding in each allosteric site and explore how differences between transporters could be exploited to generate NSS specific compounds with an emphasis on GlyT2 modulation.

## Introduction

Neurotransmitter sodium symporters (NSS) are secondary active transporters that regulate synaptic concentrations of neurotransmitters via reuptake into surrounding glial cells or presynaptic terminals. Members of the solute carrier 6 (SLC6) family act on a broad range of neurotransmitter substrates: glycine (GlyTs), dopamine (DAT), serotonin (SERT), noradrenaline (NET) and γ-aminobutyric acid (GABA, GAT) (Amara and Kuhar, 1993; Kristensen et al., 2011). Impaired functions of these transporters have been implicated in a variety of neurological disorders including addiction, depression, epilepsy, hyperekplexia, neuropathic pain, Parkinson’s disease, and schizophrenia (Benarroch, 2011; Benarroch, 2013). The role of NSS in the etiology of these conditions highlights the importance of understanding the structure-function dynamics of this family and how they can be modulated for therapeutic purposes.

The mechanism of transport has largely been inferred from crystal structures of the *Aquifex aeolicus* bacterial leucine transporter (LeuT) and supplemented by recently solved structures of the eukaryotic *Drosophila melanogaster* dopamine transporter (dDAT), human serotonin (hSERT) and human glycine transporter type 1 (hGlyT1) (Yamashita et al., 2005; Penmatsa et al., 2013; Wang et al., 2015; Coleman et al., 2016; Shahsavar et al., 2021). Substrate transport is coupled to electrochemical sodium and chloride gradients and involves substantial conformational changes that transition the transporter from an outward-facing to inward-facing state (Kristensen et al., 2011). Crystal structures of these transporters have revealed that a range of different transport modulators stabilize certain conformational states by binding in the substrate or allosteric sites. There has been a relatively recent shift in the way that drugs are designed to modulate transporter function with a greater emphasis being placed on the development of allosteric modulators of transport. Allosteric modulators are structurally dissimilar to endogenous ligands and occupy unique binding sites, minimizing the risk of side effects due to enhanced specificity (Niello et al., 2020). Currently there are no inhibitors of glycine transport used clinically despite their promising pre-clinical results as neuropathic pain analgesics (Vandenberg et al., 2014; Cioffi, 2018). This is likely due to a combination of poor pharmacokinetics and inappropriate pharmacodynamics leading to severe adverse side effects *in vivo* that highlight the necessity of further optimization of these compounds. In this review the allosteric sites identified on NSS will be explored with emphasis on both their similarities and differences across the family and how this may be exploited for drug design. We will briefly review the overall structure of these transporters and then discuss a range of ligands and how they bind to various sites. Whilst the principles will be derived from various family members, we will focus on the glycine transporter GlyT2 as a drug target.

### Architecture and Mechanism of Neurotransmitter Sodium Symporters

The structure and function of SLC6 neurotransmitter transporters has been extensively reviewed elsewhere (Forrest and Rudnick, 2009; Kristensen et al., 2011; Navratna and Gouaux, 2019) and thus will only be briefly described here. NSS consist of twelve transmembrane α-helices (TMs) that are pseudo-symmetrically arranged with respect to the membrane and are connected via a series of intracellular and extracellular loops (Yamashita et al., 2005; Krishnamurthy et al., 2009; Shi, 2013). The transport process is proposed to occur via an alternating access mechanism in which substrate and co-transported ions are exposed to either the extracellular or cytoplasmic side (Forrest et al., 2008). During translocation from the outward-open to inward-open state the core domain (TM1, TM2, TM6, and TM7) undergoes substantial conformational changes while the scaffold domain (TM3, TM4, TM8, and TM9) remains stable (Krishnamurthy and Gouaux, 2012). Ion and substrate binding initiate movement of extracellular loop 4 (EL4) into the extracellular vestibule followed by tilting and unwinding of TM5, resulting in a close association of TM1b and TM7 with EL4 that closes the extracellular gate (Krishnamurthy and Gouaux, 2012; Chen and Chung, 2015; Cheng and Bahar, 2015; Shahsavar et al., 2021). TM1a then tilts into the membrane, opening the intracellular vestibule allowing release of substrate and ions into the cytoplasm followed by a subsequent reversion to the apo-state (Krishnamurthy and Gouaux, 2012; Kazmier et al., 2017).

### Substrate Specificity and Ion Coupling of GlyTs

**FIGURE 1:**
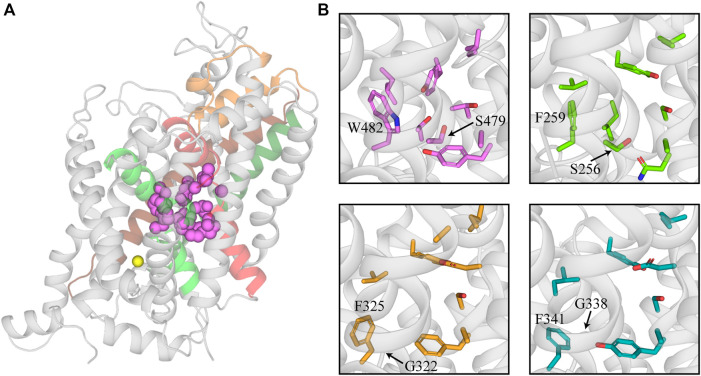
Structure of the substrate binding site of GlyT2 and comparison with other members of the NSS family. **(A)** GlyT2 homology model generated from the dDAT structure (PDB: 4M48). Residues that interact with glycine in the S1 site are represented as pink spheres. Sodium bound in Na3 of GlyT2 is shown as a yellow sphere. **(B)** Comparison of GlyT2 substrate site residues (pink sticks), LeuT (green sticks, PDB: 2A65) and NSS transporters with an outward facing atomic structure dDAT (yellow sticks, PDB: 4M48) and hSERT (teal sticks, PDB:5I73). W482 and S479 are highlighted as they restrict the size of the substrate site in GlyT2. The corresponding residues in LeuT, dDAT, and hSERT are labelled for comparison.

The substrate site (S1) of the SLC6 family is located approximately halfway across the membrane and is formed by TMs 3 and 8 and the unwound regions of TMs 1 and 6 (Figure 1A; Noskov, 2008; Piscitelli et al., 2010; Krishnamurthy and Gouaux, 2012). This hydrophobic pocket contains several non-conserved residues across the family that confers different substrate specificities for each transporter (Noskov, 2008; Wang et al., 2015; Carland et al., 2018). GlyTs have a high degree of specificity for glycine, the smallest amino acid, which is attributed to their smaller S1 site. The bulky W482 residue in GlyT2 (W376 in GlyT1) restricts the volume of S1 and sterically hinders the binding of larger substrates (Carland et al., 2018; Shahsavar et al., 2021). The corresponding residue is a phenylalanine in LeuT, SERT, and DAT, and a leucine in GAT-1, which creates a wider and less restrictive site that can accommodate larger substrates like leucine, serotonin, dopamine and GABA (Figure 1B; Beuming et al., 2006; Huang and Zhan, 2007; Noskov, 2008; Kaufmann et al., 2009). The W482F mutation in GlyT2 allows the transport of several amino acids including L-alanine and L-leucine (Carland et al., 2018). A triple mutant of LeuT that included the reverse mutation F259W produced a transporter with affinity for glycine that is similar to leucine (Noskov, 2008). In addition to differences in substrate specificity between GlyTs and other NSS, there are also differences between the two GlyT subtypes. Glycine is the only known transportable substrate of GlyT2 whereas GlyT1 is capable of transporting N-methyl-glycine (sarcosine) and N-ethyl-glycine. This has been attributed to the S479 residue in GlyT2 that is replaced by a glycine residue in GlyT1. The GlyT2 S479G mutation introduces sarcosine transport due to a less restrictive S1 site (Vandenberg et al., 2007; Werdehausen et al., 2012; Carland et al., 2018; Shahsavar et al., 2021).

There has been debate over whether there is a second substrate site (S2) located in the extracellular-facing vestibule above the S1 site. In LeuT, dissociation of sodium from the second sodium site (Na2) allosterically modulates the transporter, allowing cytoplasmic release of leucine from S1 (Terry et al., 2018). It has been suggested that when sodium is absent from Na2, leucine is able to bind S2 and trigger the intracellular release of leucine from S1 (Shi et al., 2008). Further exploration of the S2 site indicated that substrate binding to both S1 and S2 is required for the opening of the intracellular gate and subsequent release of substrate from S1 (Zhao et al., 2011). These conclusions were drawn from functional studies of a LeuT S1 mutant, F253A, which did not bind leucine but have since been disputed by structural studies showing leucine bound in S1 of this mutant (Wang and Gouaux, 2012). Structural, functional, and molecular dynamics (MD) experiments suggest that LeuT has a single high affinity substrate binding site (Piscitelli et al., 2010; Wang and Gouaux, 2012; Grouleff et al., 2017). Whilst there is a possibility that S2 is a low affinity binding site, MD simulations predict that substrate bound in S2 prevents effective closure of the extracellular vestibule, impeding, rather than facilitating, substrate translocation (Grouleff et al., 2017). However, it is important to note that experimental conditions can contribute to an obscured S2 site (Quick et al., 2012). In addition to studies on LeuT, the relevance of S2 binding has also been explored in NSS family members with evidence that S2 binding is required for the intracellular release of substrate from S1 in DAT (Shan et al., 2011). MD studies have also explored S2 in a GAT-1 homology model and suggest that GABA binding at S2 does not allosterically modulate GABA in S1 but instead, transient interactions at S2 guide GABA towards S1 (Skovstrup et al., 2012). The importance of S2 in GlyT2 has also been investigated and there was no evidence suggesting S2 facilitates substrate release from S1 (Carland et al., 2018).

As highlighted above, the transport mechanism of NSS members is dependent on the co-transport of Na^+^ and Cl^−^. SERT, DAT, and GlyT1 couple the transport of substrate with the co-transport of 2Na^+^ and 1Cl^−^ (Forrest et al., 2007; Perez-Siles et al., 2011). GlyT2, and reportedly GATs, are among the few transporters in the SLC6 family that co-transport 3Na^+^ and 1Cl^-^ with their substrate (Roux and Supplisson, 2000; Willford et al., 2015; Subramanian et al., 2016). The S1 site lies near the ion binding site of the NSS and several amino acids, including S479, interact with both substate and co-transported ions (Perez-Siles et al., 2011; Subramanian et al., 2016). The sodium one (Na1) site is highly conserved across the NSS family whereas the sodium two (Na2) site exhibits greater variance in coordinating residues (Perez-Siles et al., 2011). The third sodium site (Na3) identified in GlyT2, is coordinated by residues in TM10 (E648), TM3 (W263 and M276) and utilizes backbone interactions with TM6 (A481) (Subramanian et al., 2016). Mutating E648 in GlyT2 to the corresponding residue in GlyT1 (E648M) alters the charge to flux ratio to that of GlyT1 suggesting the negative charge of E648 is required for Na^+^ coordination in Na3 (Subramanian et al., 2016; Benito-Munoz et al., 2018). Identification of the Cl^−^ binding site initially relied on mutagenesis and modelling studies as unlike eukaryotic NSS, substrate transport by LeuT is chloride independent (Forrest et al., 2007; Zomot et al., 2007). However, resolution of the hSERT structure has supported these studies and shown Cl^−^ binding is stabilized by residues in TMs 2, 6, and 7 (Coleman et al., 2016; Coleman et al., 2020). In LeuT, the negatively charged E290 prevents binding of Cl^−^ whereas in NSS there is a conserved serine residue at this position (S513 in GlyT2) that mediates this interaction. Mutating this residue in GAT1 (S331D/E), GAT4 (S340E), DAT (S375E), and GlyT1 (S339D/E) results in a loss of chloride dependence (Zomot et al., 2007; Zhang et al., 2021).

### Vestibule Allosteric Site

**FIGURE 2:**
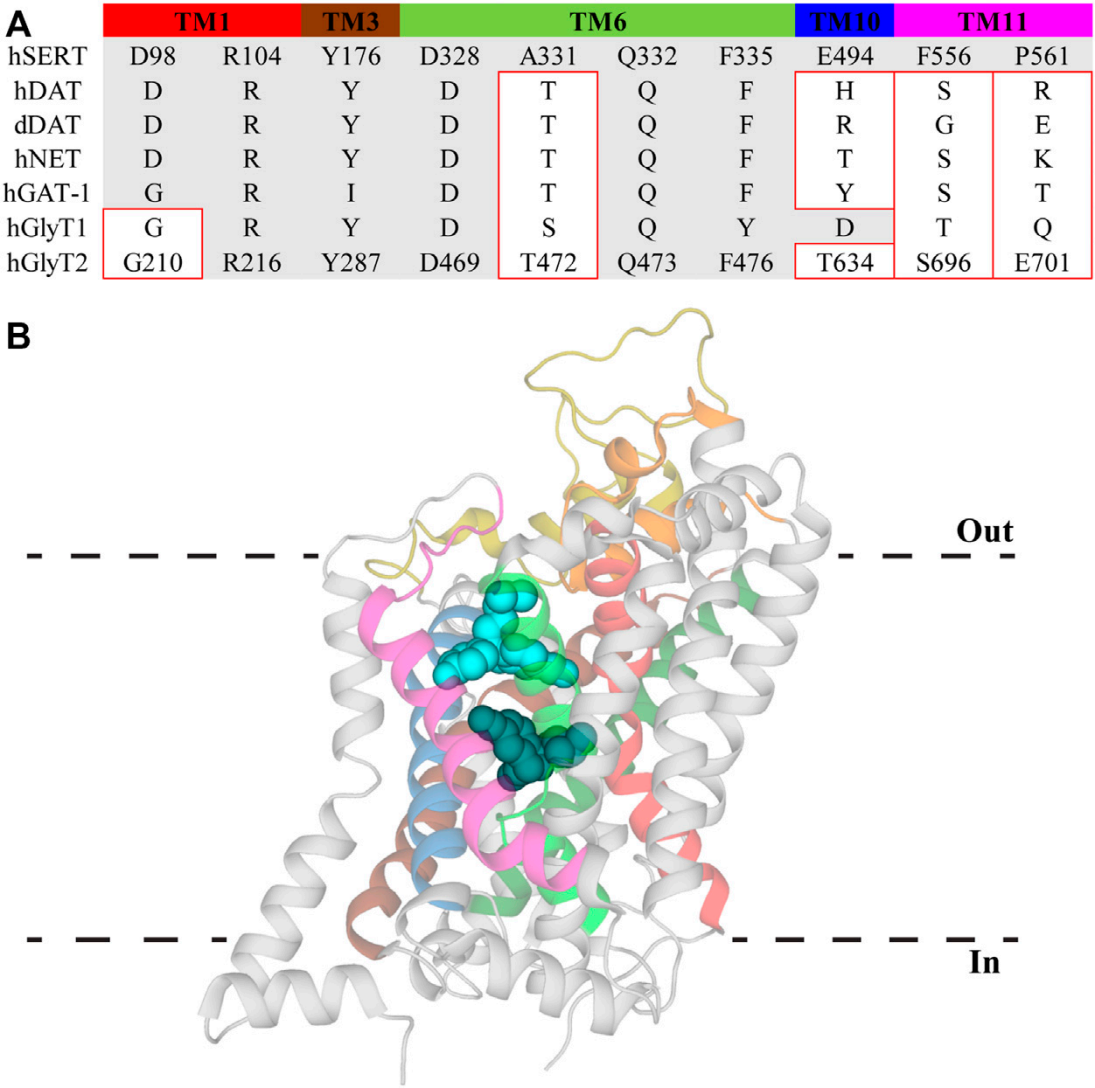
Vestibule allosteric site (VAS) in hSERT. **(A)** Sequence alignment of VAS residues in hSERT with other NSS family members. Residue numbers for GlyT2 are included to allow direct comparisons. Residues with different side chain properties are indicated with red boxes. **(B)** hSERT (PDB: 5I73) with (S)-citalopram bound in the S1 site (dark teal) and the VAS (light blue). Transmembrane domains that comprise the VAS include TM1 (red), EL2 (gold), TM3 (brown), EL4 (orange), TM6 (bright green), TM8 (dark green), TM10 (blue), and TM11 (magenta).

An allosteric modulatory site in NSS was first proposed by Wennogle et al. (1981) and its existence was supported by observations that various antidepressant drugs slow the dissociation of high affinity ligands (Chen et al., 2005; Plenge et al., 2007; Plenge et al., 2012). This site was initially resolved in crystal structures of LeuT in which tricyclic antidepressants (TCAs) and selective serotonin reuptake inhibitors (SSRIs) were bound in a region separated from the substrate site by the extracellular gate (Figure 2) (Singh et al., 2007; Zhou et al., 2007; Zhou et al., 2009). The location of this site in the extracellular vestibule has led to it being termed both the allosteric site (AS) and vestibule allosteric site (VAS), the latter of which will be used herein (Mostyn et al., 2019b; Navratna and Gouaux, 2019). In each of these LeuT structures, leucine remains stably bound in the substrate site suggesting antidepressant inhibition of LeuT occurs solely through allosterism. Given high sequence homology between LeuT and eukaryotic NSS, VAS conservation across this family was generally expected. However, antidepressants bind in the VAS of LeuT with micromolar affinities which does not correspond with the nanomolar potencies exhibited at eukaryotic transporters. This, in combination with their ability to modulate ligand dissociation, indicated the presence of a high affinity binding site on eukaryotic NSS. Radioligand displacement from hSERT by an array of antidepressants demonstrate they exhibit a competitive mechanism of inhibition (Apparsundaram et al., 2008). Furthermore, mutagenesis in this region of SERT, NET and DAT significantly alters the activity of antidepressants and psychostimulants (Beuming et al., 2008; Andersen et al., 2011; Sørensen et al., 2012). This data indicates that the orthosteric, or S1, site in eukaryotic monoamine transporters mediates high affinity binding of antidepressants and drugs of abuse. This binding mode has been confirmed with atomic structures of dDAT clearly showing these compounds occupying the orthosteric site (Penmatsa et al., 2013; Wang et al., 2015). In these structures there are no densities observed in the VAS, casting doubt over its functional relevance in eukaryotic transporters. However, recent structures of hSERT resolved (S)-citalopram in both the orthosteric S1 site and VAS, coordinated by TMs 1b, 6a, 10 and 11, as well as EL4 and EL6 (Coleman et al., 2016). Occupation of the VAS by (S)-citalopram is proposed to sterically prevent dissociation from the orthosteric site (Coleman et al., 2016). This agrees with functional studies that found (S)-citalopram bound in the VAS enhances the overall activity of this molecule while mutations in this site reduce this effect (Chen et al., 2005; Plenge et al., 2007; Plenge et al., 2012; Matthäus et al., 2016). Mutations of this site have also been shown to alter the selectivity of TCAs. Desipramine inhibits all monoamine transporters, however it demonstrates selectivity in the order NET > SERT > DAT (Eshleman et al., 1999). Desipramine potency is increased for SERT and DAT following introduction of the corresponding VAS residues in NET (Zhou et al., 2007). Currently there are no atomic structures of NET and thus no conclusive evidence regarding the functional importance of the VAS in NET is available, although this data does suggest a similar mechanism of modulation to SERT. Additionally, these results demonstrate differences in amino acid sequence of the VAS alters antidepressant binding in this region. This is likely a result of subtle movements of TM domains and extracellular loops influencing cavity formation. Indeed, a comparison of dDAT and hSERT structures shows EL2 is longer in SERT and interacts more with EL4 and EL6, changing the shape of the VAS (Coleman et al., 2016; Navratna and Gouaux, 2019). Differences in this site have the potential to be exploited for the development of allosteric modulators targeting specific NSS with the first of these recently reported. Lu AF60097 is a selective SERT inhibitor that demonstrates approximately 9-fold higher affinity for the VAS over the orthosteric site (Plenge et al., 2020). The activity of this compound is interesting because when it was applied individually, serotonin levels were not substantially elevated but when co-applied with imipramine there is a synergistic effect that significantly inhibits serotonin reuptake (Plenge et al., 2020). Therefore, it appears that crosstalk between the orthosteric site and VAS is essential for generating the full effect of these molecules. This mechanism is advantageous as co-application of these inhibitors would enable smaller dosing of centrally acting antidepressants, minimizing their off-target adverse effects. Additionally, as Lu AF60097 is highly selective and not an effective inhibitor when administered alone, it may explain the reduced side effects observed (Plenge et al., 2020).

Targeting the VAS in GAT could also be exploited to enhance the clinical viability of GAT inhibitors. Due to its expression on GABAergic neurons in the central nervous system, development of GAT inhibitors has primarily focused on GAT-1 with these compounds showing considerable potential as anti-epileptics (Iversen and Kelly, 1975; Gadea and Lopez-Colome, 2001; Łątka et al., 2020). Translation of this potential into clinical applications using competitive inhibitors has been limited by adverse effects such as motor impairment and psychotic episodes, as well as an inability to penetrate the blood brain barrier. However one inhibitor, Tiagabine, has received clinical approval (Falch et al., 1987; Madsen et al., 2007; Zafar and Jabeen, 2018). Recently, a series of hydrazones have been identified as potent, non-competitive inhibitors of GAT-1 which are proposed to bind at an allosteric site (Hauke et al., 2018). Potential binding sites for these compounds are currently unknown as there are no atomic structures of GAT-1 or, to the best of our knowledge, there has not been any substantial exploration of allosteric binding sites on GAT-1. Further studies utilizing site-directed mutagenesis or molecular modelling using homology models may help identify the binding site(s) of what are the first allosteric modulators of GAT-1. In addition, it will be of interest to examine their ability to modulate the activity of the wide array of competitive inhibitors already available, as observed for SERT inhibitors.

ORG25543 is a full, irreversible, and non-competitive inhibitor of GlyT2 (IC_50_ = 16 nM) that is analgesic in rodent models of neuropathic pain (Caulfield et al., 2001; Morita et al., 2008; Mingorance-Le Meur et al., 2013; Cioffi, 2018; Benito-Muñoz et al., 2021). However, its clinical development has been significantly hampered due to its lethality *in vivo* resulting from irreversible GlyT2 inhibition impairing synaptic recycling (Mingorance-Le Meur et al., 2013; Vandenberg et al., 2014). Understanding the binding site of ORG25543 is critical to developing derivatives that maintain high potency whilst simultaneously introducing sufficient reversibility to avoid toxicity. A computational study modelling the interaction of ORG25543 in a GlyT2 homology model identified the VAS as a potential binding site which is supported by mutagenesis studies (Benito-Muñoz et al., 2021). However, not all mutations of this region alter the potency of inhibition. This includes mutation of F478 (F478Y), F476 in hGlyT2, which exhibits the strongest binding interaction throughout the simulations. Unfortunately, the effect of these mutations on reversibility was not examined and future studies should investigate both parameters to differentiate mediators of potency and reversibility. Discrepancy between the modelling and functional data may also be a limitation of the homology model or due to the conservative nature of the mutations tested. Whilst it is tempting to speculate that ORG25543 may be the first functional high affinity inhibitor that solely binds in the VAS, computational and functional studies cannot provide definitive conclusions and further structure-function studies are required.

### Cholesterol Modulation of Neurotransmitter Sodium Symporters

**FIGURE 3:**
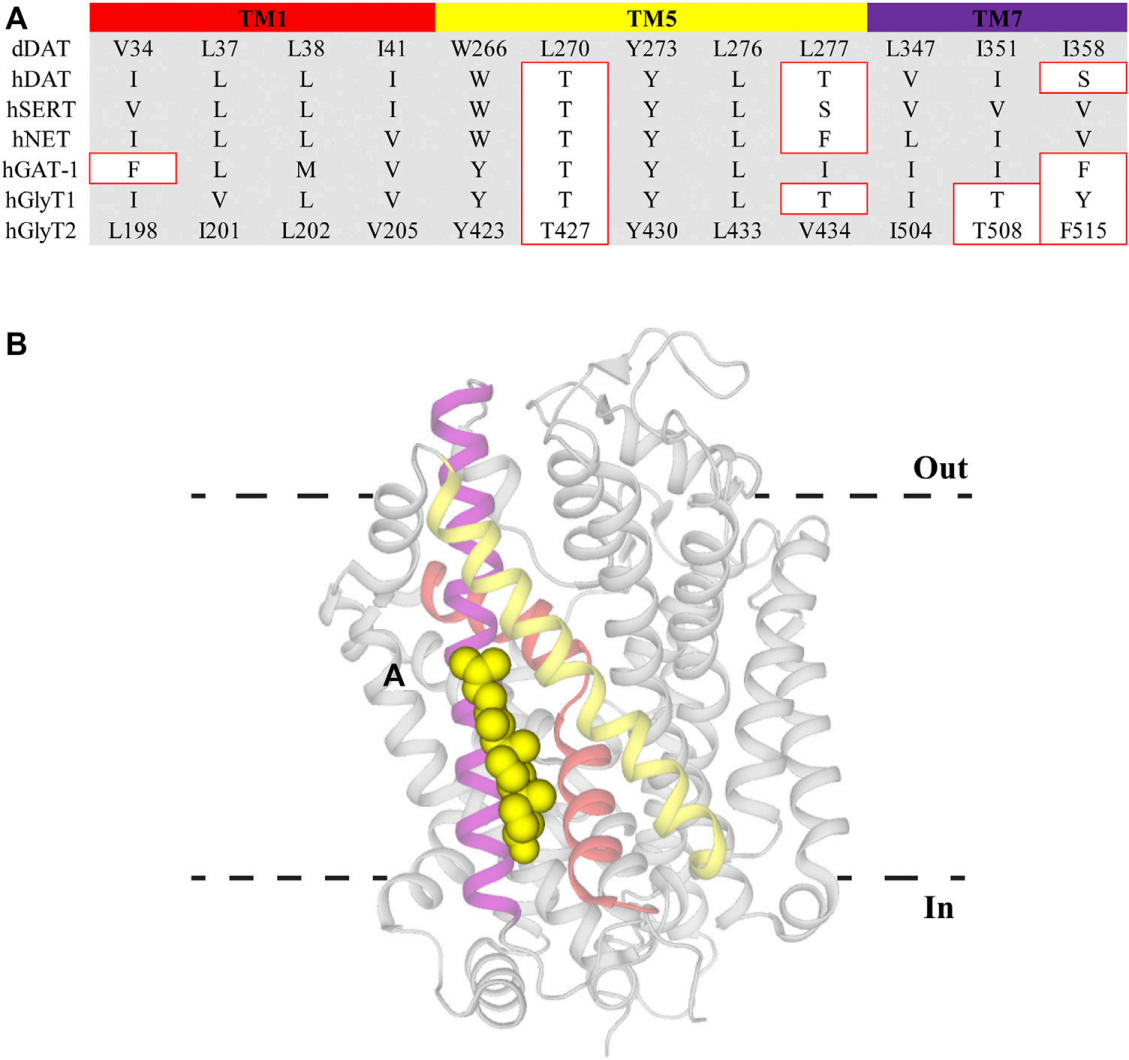
Cholesterol binding site CHOL1 in dDAT. **(A)** Sequence alignment of CHOL1 residues in dDAT with other NSS family members. Residue numbers for GlyT2 are included to allow direct comparisons. Residues with different side chain properties are indicated with red boxes. **(B)** dDAT (PDB: 4M48) with cholesterol (yellow spheres) bound in the CHOL1 site. Transmembrane domains that comprise the CHOL1 site include TM1 (red), TM5 (yellow) and TM7 (purple).

Cell membrane lipid composition and organization influence both the functionality and distribution of membrane proteins. One of the major lipid components of eukaryotic membranes is cholesterol, which can be diffusely spread throughout the membrane or concentrated in regions termed lipid rafts (Allen et al., 2007). The activity of proteins localized within lipid rafts have been reported to be modulated by the overall cholesterol content. DAT, SERT, NET, GAT-1, GlyT1, and GlyT2 have been shown to concentrate in raft sections and depletion of cholesterol using the sequestering agent methyl-β-cyclodextrin (MβCD) alters their transport kinetics (North and Fleischer, 1983; Shouffani and Kanner, 1990; Scanlon et al., 2001; Jayanthi et al., 2004; Magnani et al., 2004; Foster et al., 2008; Núñez et al., 2008; Liu et al., 2009; Hong and Amara, 2010; Jones et al., 2012). Specifically, MβCD treatment causes a reduction in the V_max_ of these transporters whilst also increasing the apparent substrate affinity of DAT and SERT. There has been debate over whether cholesterol modulation is via non-specific annular effects that alter membrane fluidity or through direct interactions of cholesterol with TM domains of the proteins (Lee, 2004). The influence of direct, non-annular, interactions has been strengthened through the observations that cholesterol and cholesterol hemisuccinate are bound at specific sites in the crystal structures of dDAT (with the binding sites termed CHOL1/2) and hSERT (CHOL3) (Penmatsa et al., 2013; Wang et al., 2015; Coleman et al., 2016). CHOL1 is formed by TM1a, TM5 and TM7 whereas CHOL2 is comprised of residues from TM2, TM7, and TM11. Both CHOL1 and CHOL2 are at a depth equivalent to the inner membrane leaflet while CHOL3 associates with the extracellular portion of TM12 (Penmatsa et al., 2013; Wang et al., 2015; Coleman et al., 2016). Within CHOL1 the α-face and iso-octyl group of cholesterol are coordinated by branched aliphatic residues whereas the β-face interacts with several aromatic residues. These coordinating residues are generally well conserved across all members of the NSS family and are found on helices that form part of the stable scaffold domain (TM1a) and flexible core domain (TM5 and TM7) indicating CHOL1 as the probable binding site for allosteric modulation of transport (Figure 3). The functional relevance of this location has been demonstrated through both site-directed mutagenesis and MD studies. Substitution of aliphatic residues to asparagine in CHOL1 generates a shift in the conformational equilibrium of hSERT to a more inward-facing state which is also observed following MβCD treatment (Bjerregaard et al., 2015; Laursen et al., 2018). This suggests that introduction of polarity in this region destabilizes cholesterol binding and alters transporter conformation. In addition, sequence analysis exploring cholesterol binding motifs and MD simulations of a hDAT homology model, based on dDAT, predicted five possible cholesterol binding sites in different TM domains (Zeppelin et al., 2018). Simulations of these sites found that cholesterol was most stably bound in CHOL1, and its presence altered the transport dynamics of hDAT by preventing disruption of interactions that mediate intracellular gating (Zeppelin et al., 2018). Together, this data suggests that cholesterol bound to CHOL1 influences the kinetics of NSS through stabilization of the outward-facing conformation. Membrane cholesterol content has also been shown to alter pharmacological sensitivity of NSS. Supplementing membranes with cholesterol increases the activity of compounds that bind the outward-facing state whereas cholesterol depletion increases potency of compounds stabilizing the inward-facing state (Hong and Amara, 2010; Laursen et al., 2018). Membrane cholesterol may also influence the functions of GlyT2. GlyT2 has been shown to be modulated by the lipid, oleoyl-L-carnitine (OLCarn) (see below). Whilst OLCarn is a slowly reversible inhibitor, co-application of MβCD with wash solution significantly speeds up the rate of recovery (Carland et al., 2013). Originally this was proposed to result from MβCD sequestrating OLCarn due to its lipid nature. However, a possible role of membrane cholesterol in modulating OLCarn interactions with GlyT2 cannot be ruled out. Future studies would benefit from pre-treating cell membranes with sequestering agents as a means of isolating the effect of membrane cholesterol. Together these studies demonstrate cholesterol clearly influences the functionality of NSS in a variety of ways and this binding site may prove valuable in fine tuning existing inhibitor activity or generating new classes of allosteric inhibitors.

### Lipid Modulation of GlyT2

**FIGURE 4:**
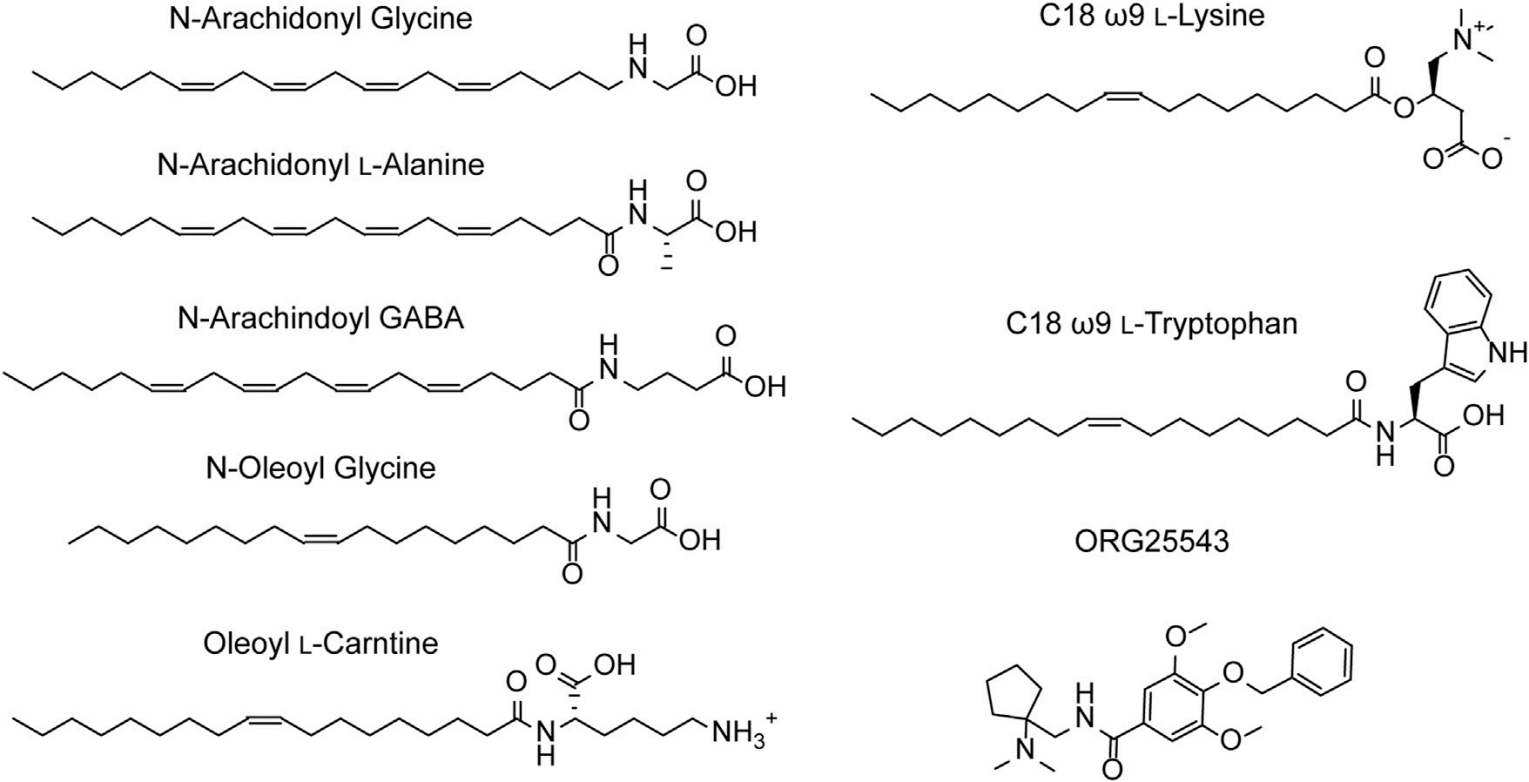
Structures of GlyT2 allosteric inhibitors.

N-arachidonyl glycine (NAGly) is an endogenous lipid with a 20-carbon (C20) polyunsaturated tail conjugated to a glycine headgroup. NAGly is most concentrated in the spinal cord and has been proposed to regulate nociceptive pathways with studies showing that intrathecal administration is analgesic in rodent neuropathic and inflammatory pain models (Huang et al., 2001; Succar et al., 2007; Vuong et al., 2008). NAGly is a partial, non-competitive, reversible inhibitor of GlyT2 (IC_50_ = 3.4 μM) and superfusion of lamina II neurons in the dorsal horn of rat spinal cord slices delays the decay of glycinergic dependent currents without altering their amplitude (Wiles et al., 2006; Edington et al., 2009; Jeong et al., 2010). Together, this data suggests that the analgesic properties of NAGly are mediated, at least in part, through inhibition of GlyT2. Identification of NAGly as a GlyT2 inhibitor prompted exploration of the inhibitory activity of other endogenous lipids including various arachidonyl amino acids and acylcarnitines. Structures of these compounds and a summary of their inhibitory activity are presented in Figure 4 and Table 1, respectively. Arachidonyl amino acids share the same polyunsaturated tail but differ in their headgroup. N-arachidonyl L-alanine inhibits GlyT2 with similar potency (IC_50_ = 8 μM) but achieves complete inhibition compared to the partial inhibition observed with NAGly (Wiles et al., 2006). N-arachidonyl GABA is less potent (IC_50_= 11.9 μM) but maintains a similar level of inhibition as NAGly (Wiles et al., 2006). Differing potencies and levels of inhibition for these compounds suggests that these properties are mediated by specific interactions between the lipids and GlyT2 rather than non-specific membrane effects. This is reinforced through MD simulations that predict addition of NAGly to a POPC/cholesterol bilayer does not alter the membrane thickness or organization of lipids (Schumann-Gillett and O’Mara, 2019). Screening of a range of acylcarnitines led to the identification of compounds with significantly improved potency compared to arachidonyl amino acids. OLCarn (IC_50_ = 340 nM) has a C18 monounsaturated acyl tail and is the most potent acylcarnitine whilst di-unsaturated or fully saturated tails do not generate substantial inhibition (Carland et al., 2013). Similarly, application of oleic acid, where the carnitine headgroup is replaced by a carboxylic acid, does not significantly inhibit GlyT2 (Carland et al., 2013). The influence of the headgroup on potency is further exemplified through the endogenous lipid N-oleoyl glycine (NOGly) where the polyunsaturated tail of NAGly is exchanged for the monounsaturated oleoyl tail. NOGly significantly improves potency (IC_50_ = 880 nM) compared to NAGly but is not as potent as OLCarn (Carland et al., 2013). Thus, both the lipid tail and headgroup are required for inhibition of GlyT2 and potency is improved by the presence of a monounsaturated acyl tail conjugated to a positively charged headgroup.

**TABLE 1:** Pharmacological profiles of select bioactive lipid inhibitors of GlyT2.

GlyT2 inhibitor	IC_50_	Reversibility	Extent of inhibition
N-arachidonyl glycine	3.4 μM^a^	Reversible^b^	Partial^b^
N-arachidonyl L-alanine	8 μM^b^	—	Full^b^
N-arachidonyl GABA	11.9 μM^b^	—	Partial^b^
Oleoyl L-carnitine	340 nM^c^	Slowly Reversible^c^	Partial^c^
N-oleoyl glycine	880 nM^c^	Reversible^d^	Partial^c^
C18 ω9 L-lysine	25 nM^e^	Reversible^e^	Full^e^
C18 ω9 D-lysine	45 nM^e^	—	Full^e^

a Edington et al. (2009).

b Wiles et al. (2006).

c Carland et al. (2013).

d Mostyn et al. (2017).

e Mostyn et al. (2019a).

Using the endogenous lipids as lead compounds a novel series of bioactive lipids that potently inhibit GlyT2 have been developed (Figure 4; Table 1). To characterize the pharmacophoric features of the acyl tail a series of acyl-glycines were synthesized that maintain the headgroup of NAGly but modify the tail configuration, double bond position and length (Mostyn et al., 2017). First, regarding carbon double bond configuration, only lipids with carbon-carbon double bonds in the *cis* configuration are active and lipids with double bonds in the *trans* configuration are inactive (Mostyn et al., 2017). Isomerization is also important for small molecule inhibitors of GlyT2, albeit to a lesser extent with only subtle differences in potency between isomers (Mostyn et al., 2020). The contrast in activity of different tail configurations suggests that the binding site of these lipids is conformationally restricted. Second, the location of the carbon-carbon double bond within the acyl tail is important as moving it two or more positions away from the ω9 position produces inactive compounds (Mostyn et al., 2017). Finally, in terms of tail length, shortening the acyl tail reduces the activity of acyl-glycines with C16 and C14 tails being less potent than C18 (Mostyn et al., 2017). This data demonstrates that the optimal acyl tail for lipid inhibition of GlyT2 is a C18 monounsaturated acyl tail with a *cis*-double bond in the ω9 position. As highlighted above, both the lipid tail and headgroup are required for, as well as influence, inhibition. In order to characterize the properties of the headgroup important for activity Mostyn et al. (2019a) conjugated a variety of amino acids to the optimal tail. Amino acid side chain properties greatly affected activity with positively charged headgroups conferring the greatest level of potency, followed by aromatic, aliphatic and negatively charged headgroups (Mostyn et al., 2019a). C18 ω9 L-lysine selectively inhibits GlyT2 with an IC_50_ of 25 nM and exhibits a mixed mechanism of inhibition (Mostyn et al., 2019a). Additionally, L-enantiomers were generally more potent, but less metabolically stable, than D-enantiomers (Mostyn et al., 2019a). Due to its metabolic stability C18 ω9 D-lysine (IC_50_ = 45 nM) was used for *in vivo* experiments and found to be analgesic in a rat model of chronic neuropathic pain, highlighting the promise of GlyT2 as a therapeutic target for analgesia (Mostyn et al., 2019a).

### Lipid Allosteric Site

**FIGURE 5:**
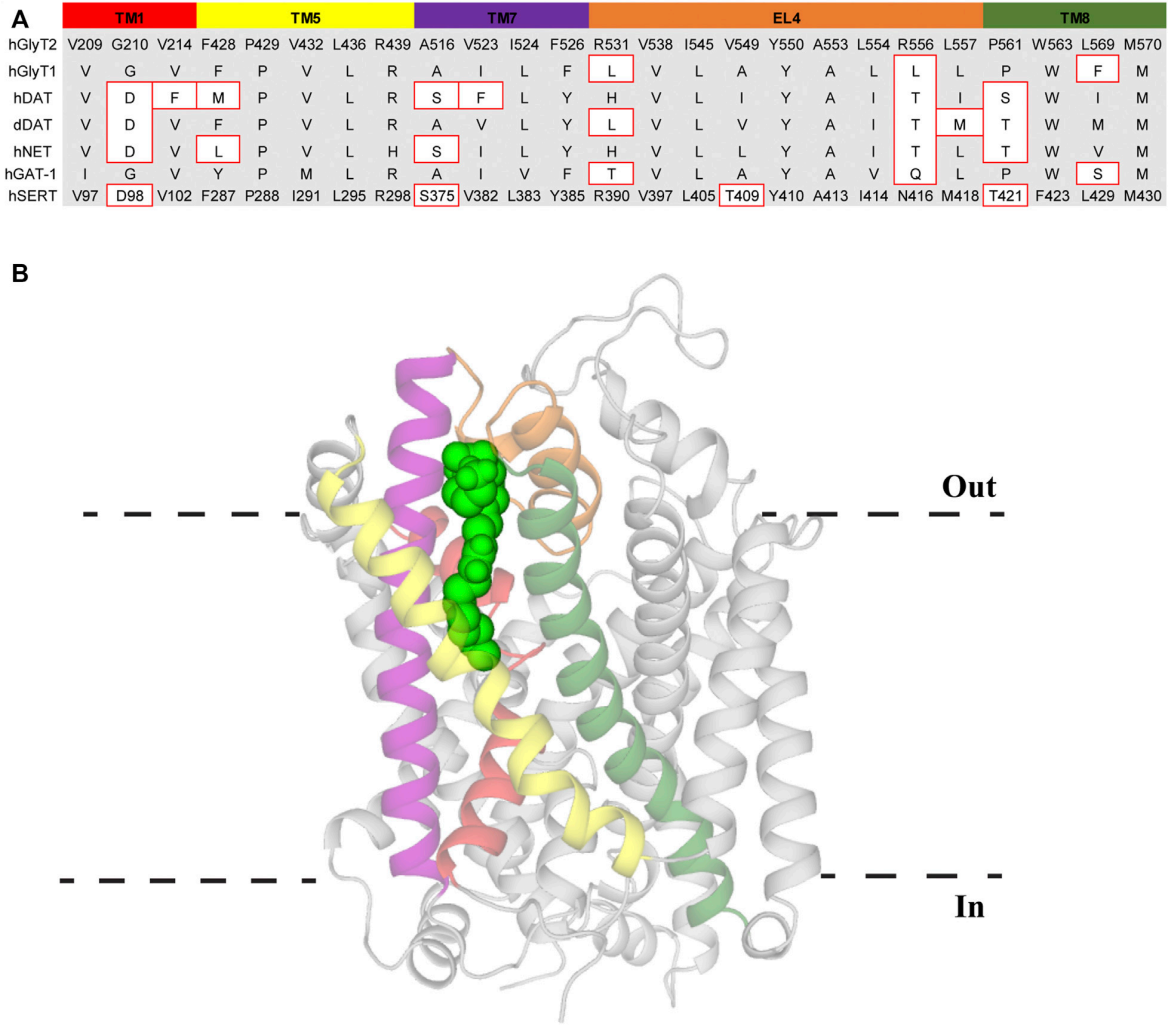
Lipid allosteric site (LAS) in GlyT2. **(A)** Sequence alignment of LAS residues in GlyT2 with other NSS family members. Residue numbers of hSERT are included for reference. Residues with different side chain properties are indicated with red boxes. **(B)** GlyT2 homology model with C18 ω9 L-lysine (bright green spheres) bound in the LAS. Transmembrane domains that comprise the LAS include TM1 (red), TM5 (yellow), TM7 (purple), EL4 (orange), and TM8 (dark green).

The high level of conservation of the VAS across the SLC6 family makes this region a promising candidate for lipid binding. However, screening of OLCarn activity on GlyT2 VAS mutants did not identify any alterations in its activity compared to the WT transporter (Mostyn et al., 2019b). Conversely, the activity of OLCarn was substantially reduced by mutations made in TMs 5, 8, and EL4 (Carland et al., 2013; Mostyn et al., 2019b). This suggests that rather than binding in the VAS, bioactive lipids bind at a novel lipid allosteric site (LAS). Simulations of compounds docked in this region of a GlyT2 homology model have been complemented with mutagenesis studies to define the LAS (Subramanian et al., 2016; Mostyn et al., 2019b; Wilson et al., 2021). During the simulations the lipids manoeuvre themselves away from their docked position such that the tail extends into a hydrophobic groove between TMs 5, 7, and 8 (Figure 5) (Mostyn et al., 2019b). Predominantly lined by aliphatic residues, access to this cavity is proposed to be influenced by I545 on EL4. Throughout all simulations the side chain of I545 was observed projecting towards the tail, inducing a kink that stabilizes binding in the hydrophobic pocket (Mostyn et al., 2019b). Despite a high conservation of the LAS between GlyT2 and GlyT1 these compounds are highly selective for GlyT2. Mutation of I545 to the corresponding GlyT1 residue (I545L) prevents the tail extending down into its binding site, forming a hairpin structure instead (Mostyn et al., 2019b). This is in agreement with functional studies of I545L mutant of GlyT2 where the inhibitory activity of C18 ω9 L-lysine is significantly reduced, and in addition, the reverse mutation in GlyT1 introduces sensitivity to C18 ω9 L-lysine (Mostyn et al., 2019b). Therefore, I545 may act as a molecular gate that restricts access to the LAS and is selective for specific tail orientations. In simulations of lipid binding to GlyT2, F428 (TM5) and L569 (TM8) engage in an inter-helical interaction that may aid in optimal formation of this hydrophobic cavity. This notion is supported by the mutation F428A reducing the activity of lipids where the double bond is in the same proximity to the headgroup, indicating alteration of the hydrophobic groove prevents accommodation of certain tails (Mostyn et al., 2019b). In initial simulations Y550 (EL4) interacts with the lipid headgroup of all compounds suggesting it interacts with a conserved element of the amino acids (Mostyn et al., 2019b). However, subsequent simulations have shown that Y550 interacts with the acyl tail double bond, and have helped explain the structure activity relationships of the bioactive lipids (Wilson et al., 2021). Y550 coordinates C18 ω9 L-lysine above the double bond whereas changing the stereochemistry of the lipid headgroup to D-lysine shifts this interaction below the double bond (Wilson et al., 2021). This shift allows Y550 to hydrogen bond with W563, locking the tail of C18 ω9 D-lysine between these two residues (Wilson et al., 2021). However, the same trend is not observed when modelling the change from C18 ω9 L-tryptophan to C18 ω9 D-tryptophan. In contrast to the lysine analogues where potency is slightly reduced, the D-tryptophan isomer is inactive (Mostyn et al., 2019a). In simulations, the tail of C18 ω9 D-tryptophan fails to properly extend into the hydrophobic groove, instead projecting towards EL4 (Wilson et al., 2021). Shallower penetration of the C18 ω9 D-lysine tail may be mitigated by stacking between Y550 and W563, resulting in a reduction in potency rather than the complete loss of activity observed with C18 ω9 D-tryptophan. This modelling data agrees with mutagenesis studies where neither acyl-lysine analogue is capable of inhibiting Y550L or W563L (Mostyn et al., 2019b). Furthermore, modelling of acyl-L-lysine analogues with shortened chain length showed an inability to insert deeply into the cavity, consistent with reduced potency observed for bioactive lipids with shorter tails (Mostyn et al., 2017; Wilson et al., 2021). Together this data suggests that deep insertion of the lipid tail into this hydrophobic cavity is essential for potent inhibition of GlyT2. Unlike the mainly aliphatic region the lipid tail occupies, the lipid headgroup is coordinated by aromatic and positively charged residues in EL4 and TM8. Lysine headgroups engage in hydrogen bonding with arginine residues (R439, R531, R556) and cation-π interactions with F526 and W563 (Mostyn et al., 2019b; Wilson et al., 2021). Tryptophan headgroups similarly interact with R439 via cation-π interactions and participate in additional π-π stacking with F526 (Mostyn et al., 2019b; Wilson et al., 2021). Stable binding of the lipid headgroup in this aromatic region may play an important role in lining up the acyl tail and assist with achieving sufficient penetration for potent inhibition.

Alignment of the LAS in the NSS family shows >80% of residues are either identical or have similar side chain properties to GlyT2 and it is therefore probable the LAS is at least partially formed in each transporter of the NSS family (Figure 5). This is supported by the conservative mutation of a leucine to isoleucine in EL4 of GlyT1 introducing bioactive lipid sensitivity and that 10 µM C18 ω9 D-lysine displaces radioligands of NET by more than 50% (Mostyn et al., 2019a; Mostyn et al., 2019b). Therefore, differences in the LAS region of the transporter may produce subtle variations of the binding pocket and ultimately this could allow the generation of novel LAS modulators that selectively target individual NSS members, potentially opening a new therapeutic avenue for some of the most clinically targeted proteins.

## Conclusion and Future Directions

**FIGURE 6:**
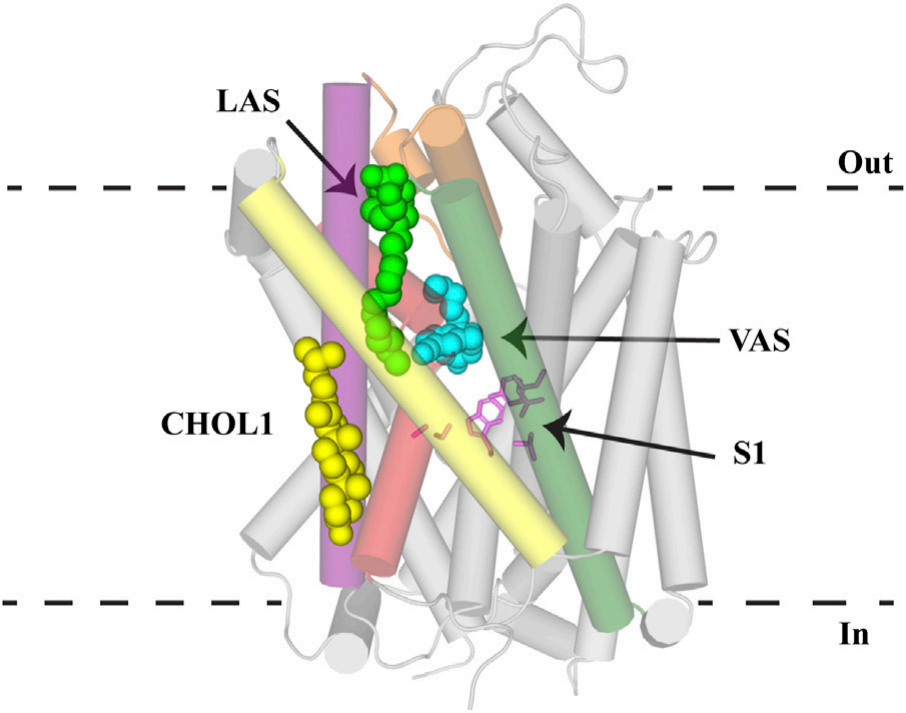
Allosteric binding sites of NSS. GlyT2 homology model with S1 residues (pink sticks) and C18 ω9 L-lysine (bright green spheres) in the lipid allosteric site (LAS). The binding location of (S)-citalopram (cyan spheres) in the vestibule allosteric site (VAS) is superimposed from the hSERT structure (PDB: 5I73). Cholesterol binding in CHOL1 is superimposed from the dDAT structure (PDB: 4M48). GlyT2 is shown as transparent cylinders with the following transmembrane domains highlighted: TM1 (red), EL4 (orange), TM5 (yellow), TM7 (purple) and TM8 (dark green).

Atomic structures of LeuT, dDAT, hSERT, and hGlyT1 have been invaluable in determining the architecture and transport mechanism of NSS. These structures, coupled with homology modelling and molecular dynamics, have identified substrate and inhibitor binding sites across this family. Understanding these sites and the interactions that mediate binding is crucial to enabling pharmacodynamic optimization of these compounds to increase their clinical viability. Three separate allosteric binding sites (VAS, CHOL1 and LAS) that modulate transport activity have been identified in NSS family members (Figure 6). High sequence homology across this family highlights the potential for these allosteric binding sites to be conserved and is exemplified through the modulatory effect of cholesterol on all transporters. Whilst there has been particular emphasis on GlyT2 as a drug target in this review, these allosteric sites are likely to be applicable to other NSS family members. Understanding of these sites is currently limited by a lack of NSS structures, including GlyT2, and that available structures of each transporter are in one conformational state. Resolution of structures of NSS in multiple conformations would enable greater understanding of how these allosteric sites fluctuate during transport and lay the foundations for developing not only novel GlyT2 inhibitors, but compounds that stabilize specific conformational states of other NSS members. It will also be important to establish whether competitive inhibitors or allosteric inhibitors will yield better clinical outcomes. The two classes of inhibitors are likely to generate different neurotransmitter clearance kinetics which may then have different impacts on neurotransmission.
